# Patterns in the Diagnosis and Management of Early Alzheimer’s Disease in Community‐Based Settings in the United States

**DOI:** 10.1002/alz.092630

**Published:** 2025-01-09

**Authors:** Timothy R Juday, Ashley A Holub, Keith A. Betts, Soeren Mattke, Sophie A Kitchen, Hongjiao Liu, Amyn Malik, Richard Batrla, Feride H Frech, Ara S Khachaturian

**Affiliations:** ^1^ Eisai Inc., Nutley, NJ USA; ^2^ Analysis Group, Boston, MA USA; ^3^ Analysis Group, Los Angeles, CA USA; ^4^ University of Southern California, Los Angeles, CA USA; ^5^ Prevent Alzheimer’s Disease 2020, Inc., Rockville, MD USA

## Abstract

**Background:**

With the changing care landscape for early Alzheimer’s disease (AD), optimizing the diagnostic and management process is key. This study assessed the correlation between patient and physician characteristics and outcomes related to the diagnosis, referral, and treatment process for early AD in community‐based settings.

**Method:**

This cross‐sectional study conducted between August and September 2023 abstracted medical chart data for patients aged 50‐89 years who were diagnosed with early AD (mild cognitive impairment [MCI] or mild AD) within the past 2 years and had a clinic visit within the past year. Generalized estimating equation models accounting for clustering at the physician level were conducted to determine the association between patient and physician characteristics and the use of biomarker/imaging assessments, referrals for cognitive/behavioral concerns, and beta‐amyloid therapies.

**Result:**

The study included 1,284 patient charts (817 MCI and 467 mild AD). Factors positively correlated with receiving biomarker/imaging assessments included younger age (<65 vs. 65+ years, adjusted odds ratio (aOR: 1.73; 95% CI: 1.22, 2.44), and physician type (neurologist vs. PCP [aOR: 2.55; 95% CI: 1.49, 4.38]). Non‐Hispanic ethnicity was also associated with lower odds of biomarker/imaging assessments (aOR = 0.58; CI = 0.37‐0.91). Patients with mild AD had increased odds of referral (aOR: 1.27; 95% CI: 1.05, 1.53) compared to patients with MCI. Younger patients (aOR: 2.82; 95% CI: 1.32, 6.06) also had increased odds of receiving beta‐amyloid therapy. While not significant, receipt of biomarker/imaging test (aOR: 2.04; 95% CI: 0.85, 4.93) and female sex (aOR: 1.44; 95% CI: 0.94, 2.21) were associated with increased odds of receiving beta‐amyloid therapy (p = ns). Likewise, patients with urban/suburban residence had increased odds of a referral (aOR: 1.31; 95% CI: 0.89, 1.94) and beta‐amyloid therapy (aOR: 1.41; 95% CI: 0.48, 4.13).

**Conclusion:**

As the care environment continues to evolve, future research should explore better measures and impact of insurance coverage policies, physician education and health care system readiness as it relates to the management of early AD, and other chronic disorders affecting memory, movement and mood.

